# Changes to the lateral geniculate nucleus in Alzheimer's disease but not dementia with Lewy bodies

**DOI:** 10.1111/nan.12249

**Published:** 2015-06-02

**Authors:** Daniel Erskine, John Paul Taylor, Michael J. Firbank, Lina Patterson, Marco Onofrj, John T. O'Brien, Ian G. McKeith, Johannes Attems, Alan J. Thomas, Chris M. Morris, Ahmad Adam Khundakar

**Affiliations:** ^1^ Institute of NeuroscienceAgeing Research LaboratoriesNewcastle upon TyneUK; ^2^ Medical Toxicology CentreWolfson Unit of Clinical PharmacologyNewcastle upon TyneUK; ^3^ Institute of Neuroscience, Biomedical Research BuildingNewcastle UniversityNewcastle upon TyneUK; ^4^ Department of PsychiatryUniversity of CambridgeCambridgeshire and Peterborough NHS Foundation TrustCambridgeUK; ^5^ Clinical NeurologyDepartment of Neuroscience and ImagingChieti ScaloItaly

**Keywords:** dementia with Lewy bodies, fMRI, lateral geniculate nucleus, neuropathology, stereology

## Abstract

**Aims:**

Complex visual hallucinations occur in 70% of dementia with Lewy bodies (DLB) cases and significantly affect patient well‐being. Visuo‐cortical and retinal abnormalities have been recorded in DLB and may play a role in visual hallucinations. The present study aimed to investigate the lateral geniculate nucleus (LGN), a visual relay centre between the retina and visual cortex, to see if changes to this structure underlie visual hallucinations in DLB.

**Methods:**

Fifty‐one [17 probable DLB, 19 control and 15 probable Alzheimer's disease (AD)] cases were recruited for a functional magnetic resonance imaging study, in which patients' response to a flashing checkerboard stimulus was detected and measured in the LGN, before comparison across experimental groups. Additionally, *post mortem* LGN tissue was acquired for a cross‐sectional study using 20 (six DLB, seven control and seven AD) cases and analysed using stereology. α‐Synuclein, phosphorylated tau and amyloid‐β pathology was also assessed in all cases.

**Results:**

DLB cases did not significantly differ from controls on neuroimaging, morphometry or pathology. However, a significant increase in amyloid‐β pathology, a reduction in number of parvocellular neurones and magnocellular gliosis was found in AD cases compared with control and DLB cases.

**Conclusions:**

These findings suggest that the early visual system is relatively spared in DLB, which implies that upstream visual structures may be largely responsible for the generation of hallucinatory percepts. The significance of the degeneration of the LGN in AD cases is uncertain.

## Introduction

Dementia with Lewy bodies (DLB) accounts for approximately 20% of demented cases at autopsy [1] and is the second most common form of degenerative dementia after Alzheimer's disease (AD) [2]. Visual hallucinations occur in 60–80% of DLB cases [3] and have been shown to have a profound effect on quality of life for patients [4, 5] and their carers [6]. Typically, the hallucinations are well‐formed and often involve people, disembodied faces, animals and objects [7].

Although other visual abnormalities frequently occur in DLB [1], the pathophysiological changes that may give rise to these phenomena remain poorly understood. Four studies using small numbers of DLB patients have shown retinal abnormalities consisting of α‐synuclein deposition [8] (challenged by Ho *et al*. [9]) and other cytoplasmic inclusions [10], retinal nerve fibre thinning [11] and electroretinogram alterations [12]. Beyond the retina, however, no evidence has yet been provided for pathological alterations in other parts of the afferent visual pathways in DLB.

Changes have also been found in the occipital lobe of DLB cases that have experienced visual hallucinations [13, 14]. Other studies employing transcranial magnetic stimulation to elicit phosphenes, an experience of seeing light in the absence of real visual input, have shown a lower threshold of stimulation in phosphene elicitation in DLB who hallucinate more frequently and severely, suggesting occipital hyperexcitation [15].

The lateral geniculate nucleus (LGN) is the first major visual processing region in the brain, and it plays a vital role in relaying information from the retinal ganglion cells, which comprise the optic nerve emanating from the retina, to the primary visual cortex [16]. Only a few studies have been conducted into how the LGN is affected under neurodegenerative conditions. Sparse tau pathology has been recorded in AD and progressive supranuclear palsy [17], whereas limited amyloid‐β pathology [18] but no Lewy body pathology has been found in DLB [19]. In light of the relative paucity of data on the LGN in DLB, the aim of this study was to investigate whether there are abnormalities in this visual structure in DLB that may contribute to the generation of complex visual hallucinations.

To carry out this study, DLB cases were compared with age‐matched control cases and ‘disease control’ AD cases. The study objectives were:

1. To investigate whether there are changes to the excitability of the LGN, by assessing functional magnetic resonance imaging (fMRI) in response to a visual stimulus and analysis of local gamma amino‐butyric acid (GABA)ergic interneurons in *post mortem* tissue.
2. To evaluate whether there are changes to neuronal and glial cell populations in the LGN across groups using stereological analysis.
3. To assess whether changes occur to neuronal sub‐populations of the LGN, using densitometric analysis of populations differentiated by calcium‐binding proteins parvalbumin and calretinin [20].
4. To investigate amyloid‐β, tau and Lewy body pathology to quantify neuropathological lesions within the LGN.

## Materials and methods

### Neuroimaging protocol

Details of participant selection, neuropsychological and neuropsychiatric assessment, and fMRI protocol have been previously reported [14]. The study was approved by the local ethics committee (Sunderland NREC). The DLB and AD diagnosis was made independently by two experienced senior clinicians (JO'B and JPT).

Participants were scanned on a 3T whole body MRI scanner (Achieva scanner; Philips Medical Systems, Best, The Netherlands). A standard whole brain structural scan (3D) MPRAGE was acquired, and functional MRI data were collected with a gradient‐echo echo planar imaging (EPI) sequence [repetition time (TR) = 1.92 s; echo time (TE) = 40 ms; field of view 192 × 192 mm^2^; matrix size 64 × 64, flip angle 90°, 27 slices, slice thickness 3 mm, slice gap 1 mm]. The stimulus consisted of five 19.2 s blocks of a circular flashing (7.5 Hz) black‐and‐white checkerboard, alternating with five 19.2 s baseline blocks of a blank screen.

Data were processed using SPM5 (Wellcome Trust Centre for Neuroimaging, University College London, London, UK) (http://www.fil.ion.ucl.ac.uk/spm/). The structural scan was segmented into grey and white matter and spatially normalised using standard segmentation. The fMRI images were aligned together to correct for motion, and spatially normalised via co‐registration with the structural scan, and smoothed with a 6 mm Gaussian kernel. For analysis with the general linear model (GLM) in SPM, a design matrix was created by convolving the onset time of the checkerboard blocks with the canonical haemodynamic response function and its first derivative. The six parameters from the motion correction were also included in the design matrix. A high pass filter of 128 s was used, and serial correlations were removed with SPM's AR(1) model. For analysis of the LGN, the ‘thalamus‐visual’ region of interest (ROI) in the SPM anatomy toolbox [21], which overlies the LGN, was used, with blood oxygen level dependent (BOLD) activity averaged across both hemispheres. For each participant, the magnitude of fMRI activation in the LGN ROI was determined by averaging the relevant beta parameter from the GLM.

### *Post mortem* tissue samples

All brain tissue was obtained from Newcastle Brain Tissue Resource (NBTR), a UK Human Tissue Authority–approved research tissue depository, and ethical approval was granted by Newcastle University ethics board and the Joint Ethics Committee of Newcastle and North Tyneside Health Authority (ref: 08/H0906/136). All subjects had been part of local prospective clinical studies and had received detailed clinical assessments during life (JPT, JO'B, AJT and IGM) and case note review after death. All cases and controls had consented to the use of their brain tissue for research purposes. Neuropathological assessment was conducted by a neuropathologist (JA) according to standardized neuropathological diagnostic procedures [1, 22, 23, 24, 25, 26]. Clinical and pathological data were collated to establish a consensus clinico‐pathological diagnosis.

Three groups of cases were included in the present study: DLB cases that had experienced complex visual hallucinations during life, AD cases that had not experienced visual hallucinations during life and aged‐control cases that showed none, or only low, age‐associated neurodegenerative pathology and had no clinical history of visual hallucinations during life. AD cases were included as disease controls with severe neurodegenerative pathology but without visual hallucinations. Cases with severe ocular pathology, such as glaucoma, were also excluded due to evidence suggesting that the LGN undergoes degeneration in these disorders [27]. Cases that had profound visual impairments were also excluded. Within the criteria specified, cases were selected based upon the availability of complete LGN tissue. The cohort used for neuropathological and morphometric analysis in this study consisted of six DLB cases, seven AD cases and seven controls (Table 1).

**Table 1:** Demographic information for *post mortem* cohort

Case ID	Age at death	PM delay	Diagnosis	Braak NFT stage	CERAD	Drug treatment
1	84	56	DLB	3	Moderate	Rivastigmine
2	89	88	DLB	3	Sparse	No
3	71	8	DLB	2	Sparse	Donepezil
4	71	68	DLB	3	Sparse	Donepezil
5	78	83	DLB	1	None	Sinemet
6	78	96	DLB	3	Sparse	Donepezil
7	78	23	Control	2	Sparse	No
8	74	45	Control	2	Frequent	No
9	85	95	Control	2	None	No
10	77	83	Control	2	None	No
11	73	25	Control	0	None	No
12	80	16	Control	2	None	No
13	85	57	Control	2	None	No
14	76	6	AD	6	Frequent	Rivastigmine
15	91	22	AD	5	Frequent	Donepezil
16	85	32	AD	5	Frequent	Donepezil
17	77	63	AD	6	Frequent	No
18	81	73	AD	5	Frequent	No
19	93	34	AD	5	Moderate	Donepezil
20	86	51	AD	6	Frequent	No

Braak NFT stage, neurofibrillary pathology stage as outlined in Braak *et al*.'s study [26]; CERAD, consortium to establish a registry of AD as outlined in Gearing *et al*.'s study [24]; PM delay, interval from time of death to autopsy.

### Neuropathology sample preparation

**Figure 1:**
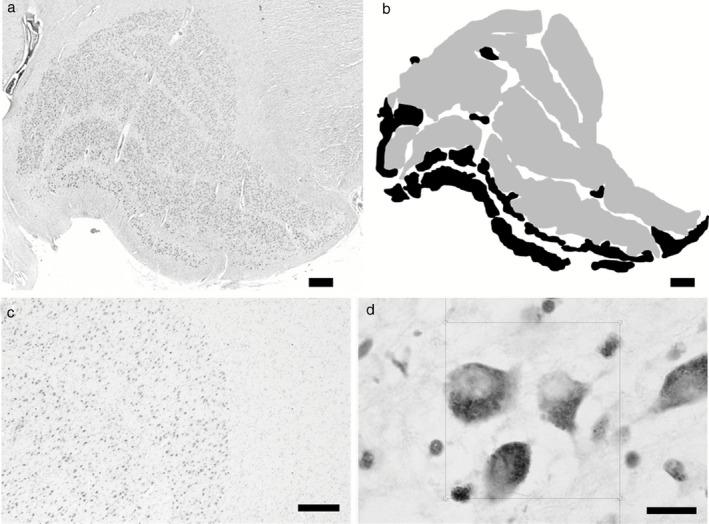
The structure of the LGN. (**a**) Shows a typical LGN approximately in the middle coronal level, with clear lamination. (**b**) Shows how the LGN was divided into magnocellular (black) and parvocellular (grey). (**c**) Shows typical LGN cells with a point grid, and (**d**) shows disector frames used to perform neuronal and glial cell counts within the LGN. Scale bars: (**a**) and (**b**) = 1 mm, (**c**) = 200 μm and (**d**) = 10 μm.

At autopsy, the right hemisphere was fixed in 10% formalin, and brain weight and *post mortem* delay were noted. Following fixation, the hemisphere was cut into 7 mm coronal slices, prior to further dissection into blocks for neuropathological assessment. From these blocks, the LGN was identified by its distinctive architecture (Figure 1 **a**) and its location on the ventrolateral surface of the posterior thalamus.

### Staining of tissues

For stereological analysis, 30 μm sections were sampled at 1 mm intervals from the entire LGN and stained with cresyl fast violet. For immunohistochemical procedures, 10 μm sections were sampled at 1.5 mm intervals from the entire LGN. Sections were incubated in the primary antiserum (5G4 anti‐α‐synuclein, Analytik Jena, Jena, Germany, 1:4500; 4G8 anti‐amyloid‐β peptide, Covance, Princeton, NJ, USA, 1:15 000; AT8 anti‐phosphorylated tau, Autogen, Holliston, MA, USA, 1:4000; anti‐GAD65/67, Sigma Aldrich, St. Louis, MO, USA, 1:12 000; anti‐parvalbumin, Sigma Aldrich, 1:8000; anti‐calretinin, Sigma Aldrich, 1:1000; anti‐calbindin, Sigma Aldrich, 1:500; anti‐calbindin, Merck Millipore, Darmstadt, Germany, 1:100). Visualization of antibody binding utilized the Menarini X‐Cell‐Plus HRP Detection Kit (Menarini, Berkshire, UK).

### Stereological analysis

Stereological analysis employed the use of a Zeiss AxioVision Z.1 microscope equipped with a motorised stage (Zeiss, Oberkochen, Germany), coupled to a computer with Stereologer software (Bethesda, MA, USA).

Measures were taken of both the magnocellular and parvocellular cell populations of the LGN. Magnocellular and parvocellular cells were characterized on the basis of location and morphology (Figure 1 **b** [28]). The laminar structure of the LGN is divided based on cellular populations, with two magnocellular ventral layers (layers 1 and 2) and four parvocellular dorsal layers (layers 3–6). As noted previously [29], ‘islands’ of magnocellular neurones exist outside of the two ventral layers of the LGN that are easily distinguished based on morphology and staining intensity with cresyl fast violet [28]. These were also included in the counts of magnocellular neurones.

To determine the number of cells in the LGN, volume was assessed using Cavalieri's principle, and cell density measures were assessed using the optical disector (as described in Mouton *et al*. [30] (see Appendix S1).

Disector frames were placed in a uniform, random arrangement (Figure 1 **c**) to calculate the density of cells within a defined region (Figure 1 **d**).

For the present study, only points that fell within the layers being investigated (i.e. magnocellular or parvocellular) were sampled for analysis (Figure 1 **b**). In anterior and posterior regions, the delineation of cell populations is complicated by the absence of corporeal lamination of the LGN. Therefore, a division was made to include cells most resembling magnocellular neurones with magnocellular analyses and those most resembling parvocellular neurones with parvocellular analyses [28].

Magnocellular and parvocellular section thickness did not significantly vary across disease groups (magnocellular *F* = 1.099, *P* = 0.356; parvocellular *F* = 1.154, *P* = 0.339). The mean coefficient of error (CE) for neuronal and glial cell estimates was calculated using the Gundersen–Jensen method [31] (see Appendix S1). CE values showed a high degree of precision, with magnocellular neurone counts having a mean CE value of 0.0663 and magnocellular glial cell counts having a mean CE value of 0.0391. Parvocellular neurone counts had a mean CE value of 0.0699 and parvocellular glial cell counts had a mean CE value of 0.0449.

### Quantification of neuropathological lesions

Images of the entire LGN were captured in sampled sections using a Nikon 90i microscope with a 20 × magnifying objective and DsFi1 camera (Nikon, Tokyo, Japan) coupled to a PC. These images were imported into the NIS elements software, and thresholds were determined separately for AT8, 4G8 and 5G4 immunoreactivity (as described by Mandler *et al*. [32]). This allowed analysis of immunopositive signals, without detection of nonspecific background staining or physiological amyloid precursor protein, as they did not reach the size nor intensity threshold for detection. The area that was immunopositive for each antibody in the LGN was recorded and a mean generated across all images taken of the LGN, from all sections per case. This gave a final result of the mean percentage area stained per case.

### Densitometric analysis of LGN neuronal populations

Images of the entire LGN were acquired in sampled sections using a Zeiss Axioplan 2 microscope (Zeiss, Oberkochen, Germany) with a 20 × magnifying objective and 3‐chip CCD true colour camera (JVC, Yokohama, Japan) coupled to a PC. These images were analysed using ImagePro Plus v.4.1 image analysis system (Media Cybernetics, Bethesda, MA, USA). Based on the techniques described in Perry *et al*.'s study [33], the mean percentage area stained on sections was determined. Each case, therefore, had a mean value generated across all sections analysed.

Despite a previous study [20] suggesting that calbindin labels koniocellular neurones, no immunoreactivity was observed in the LGN with various titrations of calbindin antibodies.

### Statistical methods

Demographic group comparisons from the neuroimaging cohorts were conducted using parametric and categorical statistics where appropriate. Group comparisons of the beta parameter magnitude of fMRI activation in the LGN ROI were carried out using analysis of variance (anova) with post hoc *t*‐tests.

To assess the differences in neuronal number, glial number, neuronal volume and densitometric data between DLB, AD and control cases, an analysis of covariance was conducted, with age at death and *post mortem* delay as covariates. Significant main effects were followed by pairwise comparison of differences, and no Bonferroni corrections for multiple comparisons were applied due to the relatively small sample size [28]. These analyses were two way and with the alpha level set at 0.05. Densitometric and neuropathological data were analysed using anova and Kruskal–Wallis tests (as appropriate) and correlated using Spearman's rho.

## Results

### Demographic variables

In the neuroimaging cohort, patients and controls were matched similarly for age, visual acuity and gender (Table 2). As expected, patients had greater cognitive impairment than controls, although AD and DLB were similarly matched for global measures (Mini Mental State Examination and Cambridge Cognitive Examination total). DLB patients had more parkinsonism, fluctuations and hallucinations but were less impaired in the memory domain compared with AD cases.

**Table 2:** Demographic, cognitive and motor characteristics of participants included in neuroimaging study

	Control (*n* = 19)	AD (*n* = 15)	DLB (*n* = 17)	anova *P*‐value
Age (years)	77.6 (7.1)	82.5 (9.2)	81.5 (5.5)	0.12
Gender (males : females)	11:8	9:6	9:8	1.00
UPDRS motor subscale	0.9 (1.6)	2.8 (3.0)	33.5 (14.4)	<0.001*^,†^
MMSE	29.0 (1.2)	20.8 (4.4)	19.0 (5.1)	<0.001*^,‡^
CAMCOG total score	96.5 (3.4)	66.5 (14.4)	64.5 (14.4)	<0.001*^,‡^
CAMCOG executive subscore	22.1 (3.4)	12.7 (5.2)	10.5 (4.9)	<0.001*^,‡^
CAMCOG memory subscore	23.6 (2.1)	10.3 (5.1)	15.4 (4.3)	<0.001*^,†,‡^
Visual acuity (decimalised)	0.64 (0.29)	0.68 (0.22)	0.58 (0.22)	0.62
CAF	–	3.0 (4.6)	7.9 (4.5)	<0.001^§^
NPI^hall^	–	0.4 (0.9)	2.9 (2.3)	0.006^§^
On cholinesterase inhibitor	–	14 (93.3)	13 (76.5)	N/A
On antiparkinson medication	–	–	–	N/A
L‐dopa dose equivalent (mg)^§^	–	–	145.8 (72.1)	N/A

*Controls *vs.* DLB *P* ≤ 0.001; ^†^AD *vs.* DLB *P* ≤ 0.001; ^‡^Controls *vs.* AD *P* ≤ 0.001; ^§^AD *vs.* DLB comparison only. DLB, dementia with Lewy bodies; UPDRS, Unified Parkinson's Disease Rating Scale; MMSE, Mini‐Mental State Examination; CAMCOG, Cambridge Cognitive Examination; CAF, Clinician Assessment of Fluctuation; NPI^hall^, Neuropsychiatric Inventory hallucinations subscale score (frequency × severity of hallucinations).

For the *post mortem* cohort, no significant differences in *post mortem* delay (*P* = 0.253) or age (*P* = 0.415) were found between groups.

### Neuroimaging results

**Figure 2:**
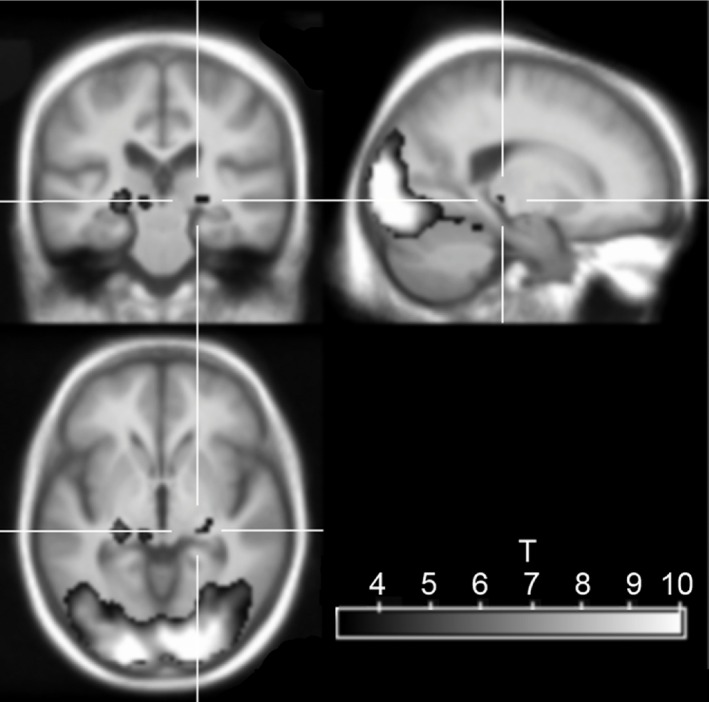
fMRI image of activation in response to checkerboard stimulus in the whole group, overlaid on an age‐matched average structural scan. Crosshair is centred on the right LGN. Yellow‐red colour scale shows the *t* statistic, thresholded at *P* < 0.001 (uncorrected for multiple comparisons).

On ROI analysis, significant BOLD activity was noted in the LGN (Figure 2) in DLB (0.24 ± 0.24; *t* = 4.22, *P* = 0.001) and controls (Beta estimate 0.16 ± 0.21; *t* = 3.18, *P* = 0.005) but failed to reach significance in AD (0.16 ± 0.35; *t* = 1.82, *P* = 0.09). However, there were no differences in activity between groups for the checkerboard stimulus in LGN (*F* = 0.54; *P* = 0.59). There were also no significant correlations between BOLD activation in the LGN and any of the clinical variables including neuropsychiatric inventory hallucinations subscale, a clinical marker for the severity and frequency of visual hallucinations.

### Stereological analyses

Results from stereological analyses are summarised in Table 3.

**Table 3:** Stereological data showing magnocellular (MG) and parvocellular (PV) neuronal and glial cell counts in the LGN

	Control	DLB	AD
MG neuron number	161 × 10^3^ ± 12 954	155 × 10^3^ ± 13 992	132 × 10^3^ ± 12 954
MG glia number	821 × 10^3^ ± 79 053	879 × 10^3^ ± 85 387	928 × 10^3^ ± 79 053
MG neuron: glial cell ratio	5.13 ± 0.319	5.74 ± 0.345	7.08 ± 0.319*
PV neuron number	131 × 10^4^ ± 89 641	141 × 10^4^ ± 96 823	111 × 10^4^ ± 89 641*
PV glia number	475 × 10^4^ ± 463 999	578 × 10^4^ ± 501 177	423 × 10^4^ ± 463 999
PV neuron: glial cell ratio	3.66 ± 0.26	4.01 ± 0.28	3.89 ± 0.26

*P < 0.05.

There was a significant main effect of diagnosis on parvocellular neurone number (*F* = 5.845, *P* = 0.013) with post hoc pairwise comparisons showing a significant 21.6% decrease in AD compared with DLB (*P* = 0.004) and a trend towards a significant 15.8% reduction in AD compared with controls (*P* = 0.064). Glial cell number differed across diagnostic groups (*F* = 4.118, *P* = 0.038) in parvocellular layers, with a significant 26.8% decrease in AD compared with DLB cases (*P* = 0.012). Magnocellular neurone and glial cell number was not significantly different across diagnostic groups.

In parvocellular layers, no significant difference in the ratio between glial cells and neurones across groups was observed. However, in magnocellular layers, a significant main effect of glial: neuronal cell number ratio across groups (*F* = 7.733, *P* = 0.005) was found, with a significant 38% increase noted in AD compared with control (*P* = 0.001) and a significant 23.3% increase in AD compared with DLB (*P* = 0.041).

Across all cases and controls, Braak stage was negatively correlated with the number of parvocellular neurones (rho = −0.473, *P* = 0.035) and the number of magnocellular neurones (rho = −0.444, *P* = 0.05).

The results of the stereological analysis showed high levels of variation in cell number between cases (Table 3). However, neither the duration of cognitive decline nor the age at onset of clinical symptoms was significantly correlated with magnocellular or parvocellular neuronal or glial cell number.

### Neuropathology

**Figure 3:**
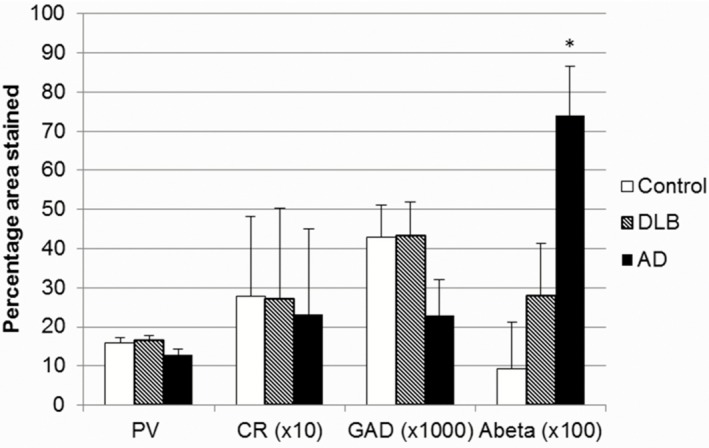
Bar chart showing percentage area stained by antibodies in the LGN across groups (*P = 0.005).

All cases exhibited an absence of both Lewy body and tau pathology within the LGN, apart from occasional sparse AT8‐immunoreactive threads in Braak stage 6 AD cases. 4G8 immunoreactivity, as an indicator of amyloid‐β pathology, was present in all cases in varying degrees and was primarily situated in the parvocellular layers of the LGN. anova suggested a significant main effect of diagnosis upon percentage area stained for amyloid‐β (*F* = 7.336, *P* = 0.005), with a significant 790% increase in amyloid‐β density observed in AD (Figure 3) when compared with controls (*P* = 0.004), whereas no respective significant difference was seen between DLB and control, and a trend towards a significant difference was seen between AD and DLB (*P* = 0.055; Figure 3). Across all cases and controls, amyloid‐β was significantly positively correlated with Braak stage (rho = 0.574, *P* = 0.007).

### Densitometric analysis of GABAergic interneurones and neuronal populations in the LGN

GAD65/67 was used to identify GABAergic neurones present within the LGN. The percentage area of GAD65/67 immunoreactivity (Figure 3) did not differ significantly between DLB (0.43 ± 0.008), control (0.43 ± 0.009) and AD (0.23 ± 0.009; Figure 3). However, GAD65/67 percentage area was significantly correlated with parvalbumin immunoreactivity (rho = 0.58, *P* = 0.006), suggesting that interneurone populations increase linearly with the projection neurone populations.

Calcium‐binding proteins were used to identify neuronal subpopulations present within the LGN [20]. Parvalbumin percentage area immunoreactivity (see Figure 3) did not significantly differ between DLB (16.28 ± 1.4), control (15.99 ± 1.2) and AD (12.98 ± 1.3). Calretinin percentage area stained (see Figure 3) also did not significantly differ between DLB (2.69 ± 0.23), control (2.79 ± 0.2) and AD (2.33 ± 0.22). However, parvalbumin percentage area was negatively correlated with Braak stage (rho = −0.562, *P* = 0.008) in all cases and controls.

## Discussion

The present study revealed similar levels of BOLD activity in the LGN ROI across groups, although in AD a significant BOLD response could not be detected. Stereological analysis showed no changes in the number of neurones in the LGN between aged‐control and DLB cases using *post mortem* tissue. However, in AD, the severity of gliosis in the magnocellular layers was significantly higher than in DLB and controls. These changes were accompanied by a moderate loss of parvocellular neurones in AD compared with DLB cases, and increased amyloid‐β pathology was found in AD cases.

There is a paucity of work describing how the LGN is affected in degenerative dementia disorders, particularly DLB. However, the visual symptomatology of DLB, which includes hallucinations and delusional misidentification [1], suggests that there may be pathological changes in the visual system that influence these phenomena.

Despite previous findings showing occipital changes in DLB cases using functional neuroimaging, the present study revealed no significant difference between DLB and control cases in the LGN. This suggests that physiological changes to the visual system that may promote hallucinations in DLB do not occur through alterations to the LGN. A weakness of the present study is that the fMRI was done on a different set of subjects to the *post mortem* sample, which precluded direct comparison of alterations in fMRI activity with findings from stereological and pathological analyses. In addition, the resolution of the fMRI is large compared with the size of the LGN, and a standard ROI was used to determine LGN activity. Thus, it may be that in the AD group, greater global atrophy could have resulted in less accurate placement of the ROI, which may explain the difficulty in detecting a significant signal in the AD group. However, this is unlikely to have affected the finding of no significant difference in activity between the control and DLB groups.

As previously reported [18], an increase in amyloid‐β pathology was found in the LGN in AD cases. However, the general absence of tau pathology in the LGN, contrasted with a previous report [17], but was consistent with another [18]. The absence of Lewy body pathology was also consistent with previous work [19]. Although the amyloid‐β burden was high in some DLB cases, it was typically indistinguishable from control cases, and no overall significant difference was observed between DLB and control cases. Braak stage was also found to be positively correlated with the amount of amyloid‐β in the LGN and negatively correlated with the number of magnocellular and parvocellular neurones and the percentage area of parvalbumin immunoreactivity. This suggests that the degree of degeneration in the LGN may be related to global neurofibrillary pathology burden.

Although previous work on the visual system in DLB has revealed retinal abnormalities [8, 10, 11, 12], the relative sparing of the LGN in DLB shown in the present study is unprecedented. It is tempting to speculate that abnormal retinal input, processed by an intact LGN, may play a role in the pathophysiology of visual hallucinations in DLB. Normal processing of abnormal retinal input could lead to the distribution of fragmented or distorted visual information, which requires completion, to visual cortical areas. In AD, where LGN abnormalities were found, and retinal abnormalities have been described [11], altered LGN processing may act as a gating function and avoiding cortical processing of altered perceptions.

The finding of specific gliosis in the magnocellular layers of the LGN in AD suggests potentially inflammatory conditions in this population of LGN neurones in AD. This may be related to the finding of specific magnocellular deficits in AD patients who have undergone physiological testing with electroretinograms and visual evoked potentials [34].

Neuropathological changes occur to the primary visual cortex in AD [35], whereas less pathology is typically observed in DLB [36]. As there is considerable reciprocal connectivity between the primary visual cortex and LGN [37], it is possible that the pathological changes observed in the LGN in AD, but not DLB, result from degeneration of the primary visual cortex in AD [35]. As pathology occurs in the primary visual cortex in late stages of AD [23, 26], and the LGN degeneration observed is relatively mild, it is tempting to speculate that the changes observed in the LGN are a late feature of AD pathology. However, if the changes do occur at late stages of AD, it is difficult to say how much of an influence that these changes may have upon the clinical phenotype.

As with most human *post mortem* studies of this nature, sample size was limited by tissue availability. Moreover, the strict sampling strategy demanded by stereological protocol, where whole, intact structures are required, further reduced the patient cohort. Shrinkage following formalin fixation is well understood [38] and, despite efforts to conduct this study based on unbiased principles, the unwitting introduction of bias by the differential rate of shrinkage across disease groups, while unlikely, cannot be excluded.

The present study implies that the LGN and, by extension, the geniculocortical visual pathway are relatively preserved in DLB but not AD. This preservation of the LGN suggests that the manifestation of visual hallucinations in DLB may be due to normal processing of altered visual input, upstream pathology in the visual system and/or pathological changes to brain structures elsewhere that have yet to be identified.

## Author contributions

DE performed neuropathological and stereological analysis of the LGN in the *post mortem* cohort and wrote and edited the manuscript.

JPT clinically assessed patients from both cohorts, conducted the fMRI study and conceived the idea for the fMRI study. He also assisted in preparing and editing the manuscript.

MJF conducted the fMRI study, conceived the idea for the fMRI study and assisted in preparing and editing the manuscript.

LP assisted with staining of tissue and analysis in the *post mortem* cohort, and also assisted in editing the manuscript.

MO provided insights into study design and helped prepare and edit the manuscript.

JO'B clinically assessed the patients from both cohorts, conducted the fMRI study and conceived the idea for the fMRI study. He also assisted in preparing and editing the manuscript.

IGM clinically assessed the patients from the *post mortem* cohort during life and assisted in preparing and editing the manuscript.

JA conducted neuropathological assessment of the cases from the *post mortem* cohort and assisted in preparing and editing the manuscript.

AJT clinically assessed patients, obtained funding for the project and helped prepare and edit the manuscript.

CMM conceived the idea, obtained funding for the project, and helped prepare and edit the manuscript. He also helped select the cases for use in the *post mortem* cohort.

AAK supervised this project, conceived the idea, obtained funding for the project, and helped prepare and edit the manuscript.

## Funding

Tissue for this study was provided by the Newcastle Brain Tissue Resource which is funded in part by a grant from the UK Medical Research Council (G0400074), by NIHR Newcastle Biomedical Research Centre and Unit awarded to the Newcastle upon Tyne NHS Foundation Trust and Newcastle University, and as part of the Brains for Dementia Research Programme jointly funded by Alzheimer's Research UK and Alzheimer's Society.

This study was funded by the Wellcome Trust and the NHS National Institute of Health Research Biomedical Research Unit for Lewy body dementia at Newcastle upon Tyne Hospitals NHS Foundation Trust and Newcastle University. DE is funded from the NHS National Institute of Health Research Biomedical Research Unit for Lewy body dementia, from the Yvonne Emily Mairy bequest. AAK is funded by the Alzheimer's Society. The funding source had no role in study design, data collection/analysis, the writing of the paper or the decision of when or where to publish it. The views expressed here are the views of the authors and not necessarily those of the NHS, NIHR or the Department of Health.

## Disclosures

None.

## Supporting information


**Appendix S1.** Stereology methods.
