# Supplementary material for: Changes to the lateral geniculate nucleus in Alzheimer's disease but not dementia with Lewy bodies
Source: Neuropathol Appl Neurobiol. 2015 Jun 2;42(4):366–76. doi: 10.1111/nan.12249 (PMC4913748; doi:10.1111/nan.12249)
Supplement: Supplementary file 1 — Appendix S1. Stereology methods. [file NAN-42-366-s001.docx]

**SUPPLEMENTARY MATERIAL 1: STEREOLOGY METHODS**

*Cavalieri estimation of volume*

Cavalieri’s principle is described by the following equation:

$$V:=T\cdot a\cdot\Sigma p$$

Where $T$ is the intersection distance, $a$ is the area per point, and $p$ is the sum of the number of counted points.

For estimation of volume, points were placed in a uniform random manner, with points spaced at 200 μm for magnocellular layers, and 600 μm for parvocellular layers. Those points which were on the LGN layers to be assessed were counted.

The rater (DE) traced an outline around the region of interest (i.e. magnocellular layers and magnocellular islands, or parvocellular layers) using a 2.5x objective.

*Optical disector*

The following equation was used to quantify the density of cells within a given region:

$$Nv=\frac{\sum^{p-} Q^{-}}{P\cdot V}$$

Where $Nv$ = numerical density, $p-$ = disector samples, $Q^{-}$ = *Q*-weighted number of objects counted, $P$ = total number of disectors, and $V$ = disector volume.

Neuronal counts using the optical disector were conducted at 63x oil-immersion objective. Counts of glial cells within the magnocellular layers utilized disector frames of 1850 μm^2^, whilst counts of magnocellular neurones within this space utilized disector frames of 3480 μm^2^. Within the parvocellular layer space, glial cell counts used frames of 1850 μm^2^ and parvocellular neurones used frames of 2810 μm^2^. In each case, all neurones that were encountered within the disector frames were counted to prevent the introduction of bias by a subjective assessment of neuronal types.

*Coefficient of error*

The Coefficient of error was calculated using the Gundersen-Jensen method, as illustrated by the following equation:

$${CE}^{2}=\left( \frac{\Sigma\left( I^{2} \right)}{\left( \Sigma I \right)^{2}}+\frac{\Sigma\left( {Volume}^{2} \right)}{\Sigma\left( {Volume}^{2} \right)}-\frac{2\Sigma\left( 1\cdot Volume \right)}{\left( \Sigma I\cdot\Sigma Volume \right)} \right)\cdot\left( \frac{n}{n-1} \right)$$

Where $I$ = neurones counted, $Volume$ = reference area x (sampling frame density)^2^ x section depth, and $n$ = number of fields.
